# Major atmospheric emissions from peat fires in Southeast Asia during non-drought years: evidence from the 2013 Sumatran fires

**DOI:** 10.1038/srep06112

**Published:** 2014-08-19

**Authors:** David L. A. Gaveau, Mohammad A. Salim, Kristell Hergoualc'h, Bruno Locatelli, Sean Sloan, Martin Wooster, Miriam E. Marlier, Elis Molidena, Husna Yaen, Ruth DeFries, Louis Verchot, Daniel Murdiyarso, Robert Nasi, Peter Holmgren, Douglas Sheil

**Affiliations:** 1 Center for International Forestry Research, P.O. Box 0113 BOCBD, Bogor 16000, Indonesia; 2 UPR BSEF, CIRAD, TA C-105/D, Campus international de Baillarguet, 34398 Montpellier Cedex 5, France; 3 Centre for Tropical Environmental and Sustainability Science, School of Marine & Tropical Biology, James Cook University, PO Box 6811, Cairns, QLD 4870, Australia; 4 King's College London, Department of Geography, KCL, Strand, London, WC2R 2LS, UK and NERC National Centre for Earth Observation; 5 Department of Ecology, Evolution, and Environmental Biology, Columbia University, New York, New York 10027, USA; 6 Center for Environmental Sustainability, Earth Institute, Columbia University. Schermerhorn Extension, 1200 Amsterdam Avenue New York, NY 10027-5557, USA; 7 Department of Geophysics and Meteorology, Bogor Agricultural University, Indonesia; 8 Department of Ecology and Natural Resource Management (INA), Norwegian University of Life Science (NMBU), Box 5003, 1432 Ås, Norway

## Abstract

Trans-boundary haze events in Southeast Asia are associated with large forest and peatland fires in Indonesia. These episodes of extreme air pollution usually occur during drought years induced by climate anomalies from the Pacific (El Niño Southern Oscillation) and Indian Oceans (Indian Ocean Dipole). However, in June 2013 – a non-drought year – Singapore's 24-hr Pollutants Standards Index reached an all-time record 246 (rated “very unhealthy”). Here, we show using remote sensing, rainfall records and other data, that the Indonesian fires behind the 2013 haze followed a two-month dry spell in a wetter-than-average year. These fires were short-lived (one week) and limited to a localized area in Central Sumatra (1.6% of Indonesia): burning an estimated 163,336 ha, including 137,044 ha (84%) on peat. Most burning was confined to deforested lands (82%; 133,216 ha). The greenhouse gas (GHG) emissions during this brief, localized event were considerable: 172 ± 59 Tg CO_2_-eq (or 31 ± 12 Tg C), representing 5–10% of Indonesia's mean annual GHG emissions for 2000–2005. Our observations show that extreme air pollution episodes in Southeast Asia are no longer restricted to drought years. We expect major haze events to be increasingly frequent because of ongoing deforestation of Indonesian peatlands.

Forest and peatland fires in Indonesia are a cause of major international concern because of the large GHG emissions associated with these fires^1^,^2^,^3^,^4^, and the negative impact of resulting aerosol emissions for human health, transport, tourism, economic activity in the Southeast Asian region^5^. Fires are typically lit for agricultural purposes during the regular dry season^6^, but their impacts are heightened during years of anomalously low rainfall^7^,^8^. Drought years in Indonesia occur when anomalously cold sea surface temperatures surround Indonesia and warm waters develop in the eastern Pacific Ocean (El Niño Southern Oscillation, ENSO) and in the western Indian Ocean (Positive Indian Ocean Dipole, IOD)^6^. ENSO conditions typically occur every three to seven years and result from weakened easterly trade winds in the western equatorial Pacific, allowing warm surface water to shift towards the coast of Peru in the eastern Pacific^9^. The positive phase of the Indian Ocean Dipole (IOD) is a related phenomenon that occurs when warm waters off the coast of Sumatra shift towards East Africa^10^. All major Southeast Asian haze events from 1960 to 2006 have occurred during years of anomalously low rainfall induced by ENSO and/or IOD conditions^7^. The fires of 1997-98, the year that saw the strongest recorded ENSO and IOD in the 20^th^ century, burned 9.7–11.7 million ha on Borneo and Sumatra and destroyed 4.5–6 million ha of species rich *Dipterocarp* forest (including 1.5–2.1 million on peat soils)^11^,^12^. Annual mean particulate matter concentrations reached 200 μg/m^3^ near fire sources (Southeast Sumatra and Southern Borneo), and exceeded the World Health Organization's 24-hr air quality target (50 μg/m^3^) for >50 days across Southeas Asia^13^. Estimated carbon emissions from these 1997-98 fires were 0.81–2.57 Pg, equivalent to 13–40% of annual global fossil fuel emissions at that time^2^.

In 2013, a year without regional climate anomalies, fires in Indonesia generated atmospheric pollution that exceeded the previous 1997-98 records over Singapore. These apparently anomalous 2013 fires prompted us to examine their cause and origin in greater detail. Our objectives were to: (i) examine the pollution levels generated; (ii) assess climatic conditions prior to the fires; (ii) quantify the area burned, (iii) assess prior vegetation cover and land ownership preceding the fires; (iv) estimate associated GHG emissions; (v) and consider the likelihood of such events recurring in the future.

## Results and discussion

**Figure 1:**
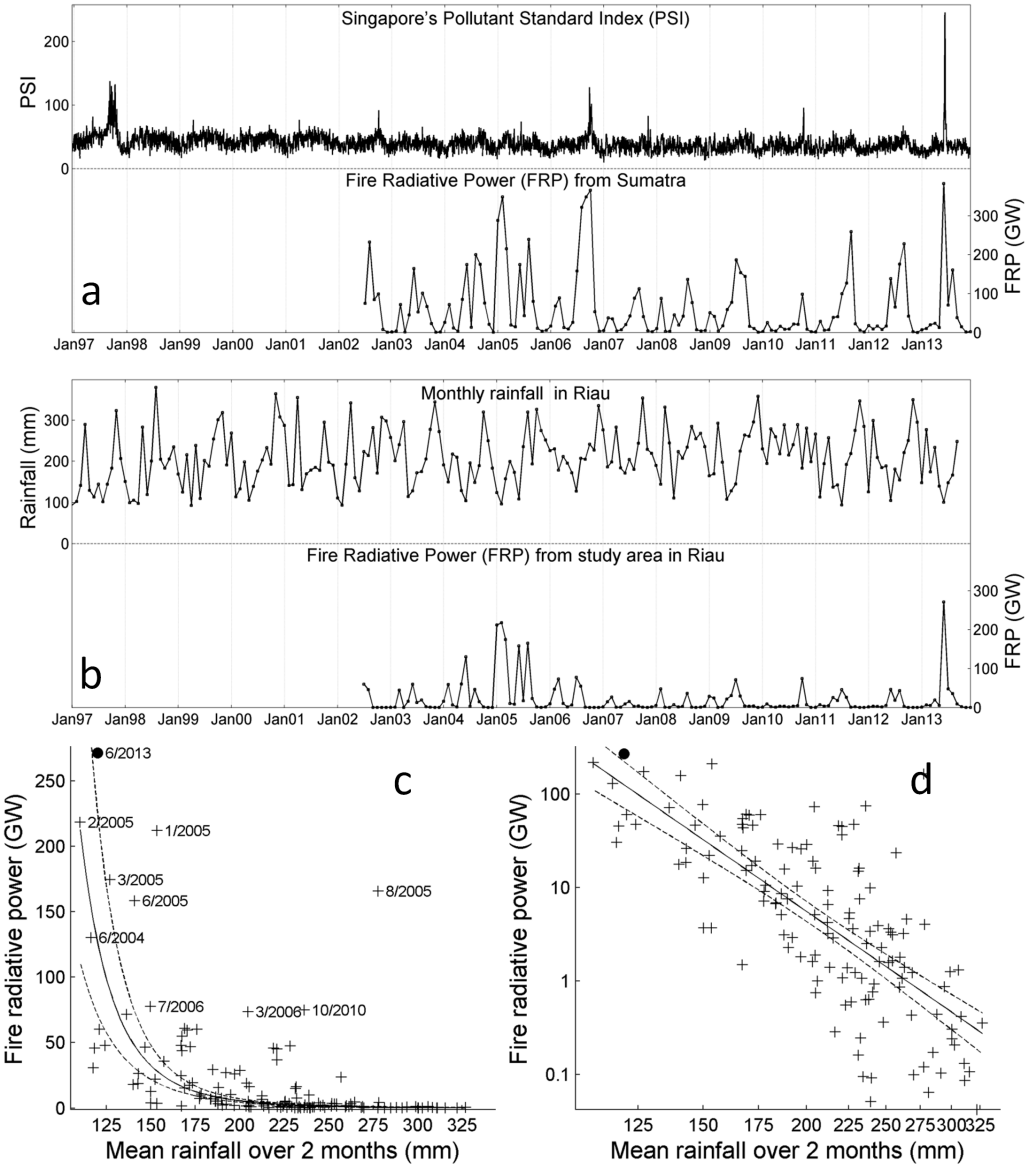
Singapore's air quality (1997–2013) and Sumatra's fire activity (2002-2013) and rainfall (1997–2013). (a), 24-hour PSI in Singapore (top) and monthly FRP (in Gigawatts) from Sumatra (bottom) measured by the MODIS instruments on-board the TERRA and AQUA satellites. (b), monthly rainfall in Riau province (top) and monthly FRP from the three-million ha study area (bottom). (c), a scatterplot of monthly FRP from the study area fitted using a power function with mean rainfall in the preceding two months. Each cross represents one calendar month (n = 134; July 2002 to August 2013), with June 2013 represented by a filled circle. (d) The same data as in c presented in Log-Log. The solid line shows the linear relation between the logarithm transformed variables: Log (FRP) = 41.1 – 6.13 Log (Rainfall). The hashed lines show the 95% prediction bounds of the fitted curve.

The largest monthly release of Fire Radiative Power (FRP) − the rate of electromagnetic energy released by fire^14^ − detected by TERRA and AQUA satellites in Sumatra (since records began in July 2002) was in June 2013 (FRP = 383 Gigawatts) (Fig. 1a). Singapore's 24-hr Pollutants Standards Index (PSI) reached an all-time record 246 on 22 June 2013 (seven consecutive days > 101 including three consecutive days > 236; rated “very unhealthy”), almost doubling its previous record of 138 from 19 September 1997 (twelve days >101 between 13 Sept. - 25 Oct.) (Fig. 1a). This trans-boundary haze event is remarkable as neither ENSO nor IOD conditions occurred in 2013. By contrast, the last major episode of extreme air pollution over Singapore had occurred in 2006, when both ENSO and IOD conditions preceded major fires in Sumatra (and in Indonesian Borneo)^15^, resulting in a peak in Sumatra's fire activity in October 2006 (FRP = 366 Gigawatts), and a peak in Singapore's 24-hr PSI on 07 October 2006 (128; rated “unhealthy”) (Fig. 1a).

**Figure 2:**
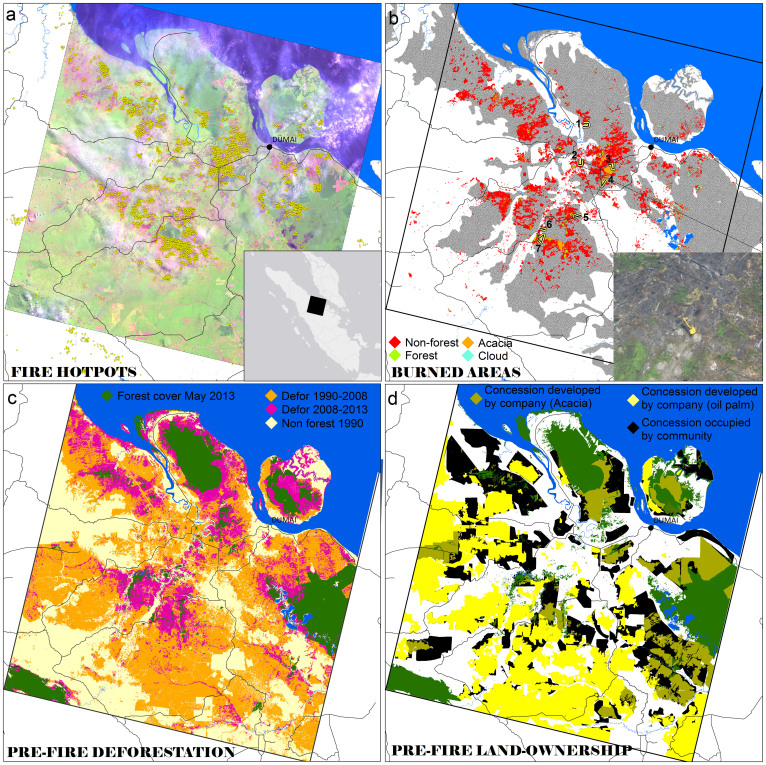
The three-million ha study area in Riau province, Sumatra (location see inset). (a), Fire hotspots. MODIS daily hotspots distribution for June 2013 (yellow dots) overlaid on a post-fire LANDSAT OLI imagery (12 August 2013) displayed in false colours (RGB: 6-5-4). (b), Burned areas. An estimated 163,336 ha burned in the study area: red (non-forest), green (forest), orange (*Acacia* plantation) and cyan (cloud). Peatlands are shown in darkest shade of grey; superimposed are the seven locations of the UAV transects. The bottom inset is a UAV snapshot over peatlands deforested 3 years prior to the June 2013 fire, where dead carbonized tree trunks and an excavator preparing land for oil palm are clearly visible. (c), Pre-fire Deforestation. Loss of species-rich *Dipterocarp* forest from 1990 until May 2013. Light brown: non forest in 1990. Orange: deforested between 1990-2008. Purple: deforested between 2008 and May 2013. The study area lost 1.72 million ha (78%) of forest between 1990 and May 2013 (including 1 million ha on peat). (d), Pre-fire land-ownership map. Industrial oil palm and *Acacia* plantations developed by companies in concessions are shown in yellow, and in khaki, respectively. Concessions (for both oil palm and *Acacia*) occupied by communities are shown in black. Lands outside concessions are in white. Forest cover (unoccupied land) one month before fire is shown in dark green. Maps created using *ArcMap* v10.0 geospatial processing program. The data used to generate the maps presented in this figure are made available online at http://www.cifor.org/map/fire/.

Our investigations of the June 2013 fires in Sumatra determined that a three-million ha area of Riau Province in Central Sumatra (1.6% of Indonesia's landmass; one LANDSAT scene; see bottom inset in Fig. 2a) was the source of 71% (271 Gigawatts) of Sumatra's FRP in that period (Fig. 1a, b). It also accounted for 72% of the area burned across the entire island as detected by the same satellites in the same month (Supplementary Fig. 1). We investigate this area in greater detail in the following sections.

Daily fire hotspots (also from TERRA and AQUA satellites) revealed a peak in fire activity during the week of 18–24 June (Supplementary Fig. 2). A brief dry period preceded this fire. For the twelve months leading up to and including June 2013 (July 2012-June 2013) Riau was wetter than average, receiving 2,530 mm of rain compared to the annual mean of 2,350 mm for 1961–2013 (Supplementary Fig. 3a). However, May and June 2013 registered rainfall deficits compared to the monthly means (Supplementary Fig. 3b). Monthly FRP in the study area was correlated with rainfall over the month of FRP measurements and the month before (Log-Log fit; r^2^ = 0.55, p<0.01, n = 134). A 1% decrease in rainfall induced a 6% increase in FRP (Fig. 1c, d). Correlations over one, three, and four months were lower (r^2^ = 0.43, r^2^ = 0.43, r^2^ = 0.31, respectively). A similar relationship has been observed previously in Central Sumatra^15^.

LANDSAT satellite imagery acquired shortly before and shortly after the fire indicates that 163,336 ha (including 137,044 ha, or 84% on peat) burned in the three-million ha study area (Fig. 2a, b). We validated this assessment using a Unmanned Aerial Vehicle (UAV or “drone”) one month after fire at seven sites, spanning 1,301 ha (Fig. 2b), and observed an accuracy of 85% for burned areas (Supplementary Fig. 4, Supplementary Table 1, 2), with 96% of MODIS fire hotspots falling within the burned areas extent (Fig. 2a, b).

**Figure 3:**
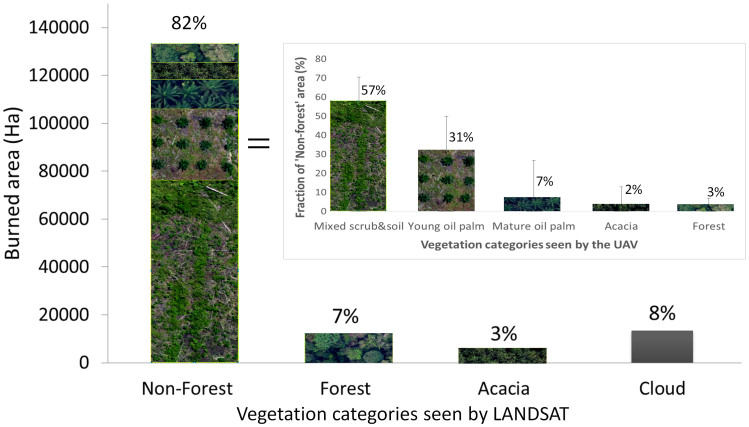
Vegetation cover of the burned areas (163,336 ha) before the June 2013 fires. The ‘Non-forest’ category contains a broad mix of vegetation types, which we identified through a comparison against a more detailed vegetation classification derived from the Unmanned Aerial Vehicle (UAV). This comparison was only performed in the portions of UAV imagery identified as ‘unburned’ (567 ha). The error bar is calculated as +-1 Standard Deviation, (n = 7 UAV transects). Inset, ‘Non-Forest’ is dominated by scrubs and exposed soils, young (<5 years old) and mature (>5 years old) oil palm plantations. Oil palm plantations either belong to small- and medium-scale agriculturalists or to companies. The ‘Forest’ category includes logged and drained natural forests. The ‘*Acacia*’ category indicates closed-canopy industrial plantations on peatlands. The ‘Cloud’ class indicates areas that were obscured by clouds and cloud shadows on the pre-fire LANDSAT imagery.

Only 7% (12,037 ha) of burned lands were classified as ‘forest’ before the fires (accuracy of 97%; Supplementary Table 3, 4). This was mainly small degraded remnants of drained peat-swamp forest. Most burned lands were classified as ‘non-forest’ (81%; 133,216 ha) (Fig. 3). However, over half of burned areas (58%; 94,308 ha) were forested five years previously (Fig. 2b, c). Comparison with the corresponding UAV-based vegetation map reveals that 57% of burned ‘non-forest’ areas were nonetheless ‘forest cemeteries’, i.e. a mosaic of scrub and exposed soil, with stumps, downed trunks and branches (Fig. 3). The burned ‘non-forest’ areas on peat (68%; 111,561 ha) generated the bulk of the FRP (Supplementary Fig. 5). The imagery also detected areas where planted *Acacia* forests (*Acacia* *crassicarpa* A.Cunn ex Benth. widely called “Acacia” though it was recently renamed *Racosperma crassicarpum* (A. Cunn. ex Benth.) Pedley.) and oil palm plantations (*Elaeis guineensis* Jacq.) had been damaged by fire (Fig. 3).

We found that 52% of the total burned area (84,717 ha) was within concessions, i.e. land allocated to companies for plantation development (Fig. 2b, d). However, 60% of burned areas in concessions (50,248 ha, or 31% of total burned area) was also occupied by communities (Methods; Supplementary Fig. 6). This presence makes attribution of fires problematic. The remaining 48% of the total burned land (79,012 ha) was owned by Indonesia's Ministry of Forestry (under central government). These areas were deforested prior to fires and their ownership is often contested by the local government. The detection of two excavators by the UAV preparing land for planting in the burned areas one month after fire suggests fires were associated with agriculture (lower inset in Fig. 2b).

We estimate that the June 2013 fires released 172 ± 59 Tg CO_2_-eq of GHG into the atmosphere during the week of 18–24 June in the study area (Table 1; Methods and Supplementary methods). Carbon emissions were 31 ± 12 Tg C. Uncertainties were around 39% and 35% of total C and total GHG emissions, respectively. These emissions represent 5–10% of Indonesia's reported annual GHG emissions for 2000–2005^16^ and 26% of average annual C emissions from fires in tropical Asia (-10 to 10N, 60–190E) between 2003–2008 modelled using the Global Fire Emission Database (GFED)^17^. Ninety percent of the emissions originated from peat and CO_2_-eq emissions were mainly in the form of CO_2_ (55.3%) and CH_4_ (44.5%). Total CH_4_ emissions represented 4–6% of average annual emission rate for the whole of Southeast Asia in 2000–2009^18^. N_2_O emissions were negligible (0.3% of total CO_2_-eq emissions).

Our results demonstrate that the Indonesian fires of 2013 behind the record air pollution episode in Singapore were triggered by a seasonal two month dry spell in an otherwise rainy year. These fires were short-lived and confined to recently deforested peatlands in a localized area in Central Sumatra (in Riau Province), reflecting ongoing conversion to oil palm plantations. The area affected was much smaller than the 9.7–11.7 million ha that burned in 1997^11^,^12^. However, the emissions of GHG and smoke during this brief localized event (one week and 1.6% of Indonesia's land) were disproportionately large because of the peat. These fires generated unprecedented atmospheric pollution in Singapore because of their proximity and the prevailing south westerly monsoon winds (Supplementary Fig. 7a).

During the last major drought years (1997 and 2006) fires peaked in Southeast Sumatra (Supplementary Fig. 7b) and in southern Borneo from August through October, but their impacts in Central Sumatra were less extreme^15^. Riau experiences a bi-modal annual rainfall pattern with peaks centred on November and April (Supplementary Fig. 2b)^19^. It responds less to sea surface temperature anomalies than Indonesia's other fire-prone regions^19^. The major Riau fires of 2005, 2013, and the recent 2014 event occurred during the regular short seasonal dry spells (< 2 months) centred on February and June. February fires (e.g. in 2005 and early 2014) are associated with prevailing north easterly monsoon winds, and thus generally cause little problem of trans-boundary haze (Fig. 1a&b; Supplementary Fig. 7c). In June, as observed in 2013, prevailing winds carry any emissions directly from Riau to Singapore (Supplementary Fig. 7a). The brief droughts, that seldom exceed two months, pose a challenge to forecasting severe fire events in Riau. While the 2013 fire event may initially appear anomalous, we expect such events to be increasingly frequent with ongoing peatland development.

Peat forests in Sumatra have declined by 18,400 km^2^ (4.6% yr^−1^) over the last two decades^20^. This reflected timber cutting, plantation development and fires^20^. Some of these deforested lands remain undeveloped and persist in a degraded and seasonally fire-prone state^21^. Deforestation elevates local temperatures, reduces precipitation and limits soil moisture; this heightens climatic variability and likelihood of drought, and influences regional climate^22^,^23^. The convergence of these trends with the frequent use of fire by humans may, over time, render the emissions of peatland fires in Central Sumatra during ‘wet’ years increasingly similar to that of ‘dry’ ENSO/IOD years. Assessing the state and vulnerability of remaining peatlands, would help identify where vigilance is most required.

The Indonesian government has encouraged investment in oil-palm and pulpwood industries resulting in rapid large-scale plantation expansion and associated developments^24^. In 2011, Indonesia implemented a moratorium on new plantation concessions in an effort to protect remaining forests and peatlands^25^. However, such policies did not prevent the June 2013 fires. Our results show that these fires occurred mostly in already-cleared peatlands. Burn locations suggest ignition by both communities and companies. Most fires are lit in order to prepare land for cultivation^24^ but some are likely accidental, while others may be arson^26^: we still know too little concerning these specific events and the intentions and safeguards used.

Efforts to avoid major haze events require that all land users control fire use during any dry periods. Given land use practices in the region, and the frequent conflicts among land users, this will be challenging^5^. We advocate active protection of remaining peatland areas and cessation of further drainage. Financial incentives for forest protection are not competitive with commercial land values, and future payments for reducing emissions from deforestation and forest degradation (REDD) are unlikely to change this^24^. Unless strong action is taken Indonesia's peatlands are likely to remain a major source of GHG and aerosol emissions.

## Methods

### Singapore's air pollution

We obtained Singapore's 24-hour Pollutant Standards Index (PSI) time series from the National Environment Agency (NEA). The PSI is a number representing the highest sub-index of five common pollutants computed based on the concentrations averaged over a 24-hour period: particulate matter (PM_10_), sulphur dioxide (SO_2_), carbon monoxide (CO), ozone (O_3_), and nitrogen dioxide (NO_2_). Systematic 24-hr PSI records began on 01 January 1997. Initially, the NEA only reported the maximum value from all ambient air monitoring stations in Singapore, but since 14 February 2005 the NEA has reported the PSI for each of the five regions of Singapore, separately, as well as the maximum value, which represents the PSI for overall Singapore. Prior to 24 August 2012, the PSI was reported once a day at 4 pm, but subsequently reports were increased to several times a day. To allow comparison for the whole time series (1997–2013) we used the 24-hr maximum PSI recorded at 4 pm when multiple values were available.

### Sumatra-wide fire activity

The MODIS satellites have been recording the rate of thermal electromagnetic energy released by fire (Fire Radiative Power, FRP) since year 2002. We combined monthly fire radiative power (FRP) from TERRA and AQUA satellites (MOD14CMH + MYD14CMH datasets^27^) to capture fire activity four times a day at 10:30 am and 10:30pm (TERRA) and at 1:30 pm and 1:30 am (AQUA). Both datasets are gridded statistical summary of MODIS fire pixel information at 0.5 arc-deg spatial resolution.

We estimated the area that had burned across Sumatra, using 500-m spatial resolution MODIS data, and specifically the MCD65A1 dataset^27^. The burned area detection method used in the MCD65A1 product was preferred over the alternative MODIS MCD45A1 burned area product (v5.1) because the former is more tolerant of cloud and aerosol contamination^28^, and the latter appeared less accurate at detecting burns in Sumatra in June 2013.

We combined 1-km^2^ daily maximum FRP (MOD14A1 +MYD14A1 products^27^) to capture daily fire activity from both the 10:30 am and 1:30 pm satellite overpasses and overlaid these 1 km^2^ FRP observations with the LANDSAT-based burned area map described in Fig. 2b to understand how fire activity varied among different vegetation cover types.

We estimated the locations of fire hotspots using the standard MOD14/MYD14 Fire and Thermal Anomalies product available at the NASA FIRMS website^29^.

### Fire and rainfall correlation

We analysed the correlation between monthly fire radiative power (FRP) and monthly rainfall from November 2000 and August 2013 in our three million study area in Riau province, Sumatra. Rainfall data came from NOAA^30^,^31^. Because droughts of different lengths can influence fires^15^, we explored the correlation between FRP and the average precipitation for up to 4 months before fire.

### Mapping burned areas and prior vegetation in the three million ha study area

We mapped burned areas and the vegetation cover of the same areas one month before the fire using three post-fire LANDSAT 8 images acquired on 25 June, 11 July and 12 August 2013 and two pre-fire images acquired on 22 April and 25 May 2013 (Supplementary Fig. 2). We employed multiple pre- and post-fire images to reduce areas contaminated by clouds and haze. In the post-fire LANDSAT imagery displayed in false RGB colour (Short-wave infrared: band 6; Near infrared: band 5; Red: band 4) unburned vegetation appear green (Fig. 2a). Pink areas reveal unburned areas with exposed soils. Burned areas appear dark red. The most severely burned areas are generally the darkest. Burned, unburned areas and pre-fire vegetation were mapped using a tree-based supervised classification algorithm. Burned areas underneath haze or clouds were digitized using visual interpretation after applying a local contrast enhancement. In the pre-fire LANDSAT imagery, forest, non-forest, *Acacia* forest industrial plantations, and clouds were mapped. We used a tree-based supervised classification method (*See5* module) in the *ERDAS Imagine* v8.6 remote sensing program to extract the burned areas and pre-fire vegetation from the LANDSAT imagery.

We collected high-resolution imagery (10-cm) with an Unmanned Aerial Vehicle (UAV) or “drone” (Skywalker Aero model with a camera Canon S100) between 28 July and 02 August 2013 to: (i) evaluate the accuracy of our LANDSAT-based maps (See Supplementary results); and (ii) characterise the vegetation types of the broad LANDSAT-based ‘non-forest’ class. The UAV images were acquired along transects at seven different sites, encompassing a variety of vegetation types and proximity to agriculture, burned and unburned mosaics (Fig. 2b). The UAV imagery (1,301 ha) was ortho-rectified and registered to our LANDSAT imagery.

We characterised the LANDSAT-based ‘non-forest’ class by first interpreting the UAV imagery into five vegetation classes: (i) scrubs and exposed soils, (ii) young oil palm, (iii) mature oil palm, (iv) *Acacia*, and (v) forest. In Riau, oil palm plantations either belong to small- and medium-scale agriculturalists or to companies. Young and mature oil palm refer to open (<5 years old) and closed (>5 years old) canopy plantations, respectively. *Acacia* indicate closed-canopy company-owned plantations on peatlands. The pre-fire LANDSAT-based ‘non-forest’ class was then defined by comparing it against the five UAV-based vegetation classes. This comparison was only performed in the portions of UAV imagery identified as ‘unburned’ (567 ha). The error bar is calculated as ±1 Standard Deviation, (n = 7 UAV transects).

To evaluate the accuracy of the LANDSAT-based ‘burned area’ map, we randomly sampled 2,088 validation points each being at least 100 m from each other. For each point, a 30 m × 30 m area, approximating a single LANDSAT pixel was visually interpreted as either ‘burned’ or ‘unburned’ in the UAV photos at 1:1,000 scale, burned areas being easily discernable (Supplementary Fig. 4). A confusion matrix determined the frequency of class agreement between our reference UAV imagery and our LANDSAT-based burned area map, as determined by overall accuracy (i.e., ‘% correct’), user's and producer's accuracy. We also identified the level of correspondence between our LANDSAT-based burned area map and the MODIS fire hotspots data by calculating the percentage of fire hotspots that fell within the burned areas or that were within 500 meters of the burned areas. We repeated this validation procedure using the portions of UAV imagery identified as ‘unburned’ (567 ha) to validate the pre-fire LANDSAT-based vegetation cover (forest, *Acacia*, non-forest).

### Mapping deforestation in the study area

We combined published LANDSAT-based datasets^32^,^33^ to map the loss of species rich *dipterocarp* forests in the study area from 1990 until 2012, and extended the analysis to May 2013 (one month before fire) using two pre-fire LANDSAT images described above. The combination of datasets involved discarding ‘tree cover loss’ pixels generated by the first dataset^33^ that fell outside of remaining forest areas in year 2000 in the second dataset^32^ to remove areas where tree loss included clearing of industrial plantations (e.g. oil palm and *Acacia*) and mixed traditional gardens (e.g. rubber, orchards, smallholder oil palm and other agro-forests mixed with forest re-growth).

### Land ownership in the study area

We obtained concession maps for 2010 at 1:250,000 scale from Indonesia's Ministry of Forestry. These concessions represent the areas allocated by the Indonesian government to companies for planting monoculture plantations of oil palm or *Acacia* (for pulpwood). Concessions (51% of our study area, or 1,661,072 ha) were disaggregated into: (i) areas developed by plantation companies (1,071,116 ha); (ii) areas occupied by small-scale agriculturalists (538,045 ha); and (iii) idle undeveloped lands (51,911 ha). This partitioning could be achieved by delineating the grid-like spatial arrangements of land parcels on the pre-fire LANDSAT imagery (Supplementary Fig. 6). This grid-like network of roads and canals on the pre-fire LANDSAT imagery is known to characterize the spatial arrangement of company-owned plantations in the Indonesian lowlands. We delimited the boundary of those grids (and in some cases concentric patterns) in a GIS by visual interpretation, and assigned them to either oil palm or *Acacia* land holdings using the publicly available concession maps. Areas in concessions that did not show grid-like patterns, but exhibited clusters of rectangular land parcels of varying shape, size, and direction were characterized as lands occupied by small-scale agriculturalists (Supplementary Fig. 6). Areas in concessions without clusters of rectangular land parcels were characterized as idle undeveloped lands (these were mainly forest remnants).

### GHG and carbon emission estimates

Fire emissions for each burning specific ecosystem/pool were calculated as the product of burned area, fuel load, combustion completeness and gas-specific emission factor. Detailed methods and references used for vegetation fuel load and combustion completeness calculation in each land use category are presented in Supplementary information. The mass of peat actually burned, i.e. product of fuel load and combustion completeness was taken from the 2013 IPCC guidelines (353 ± 186.7 Mg DM ha^−1^)^34^. The CO_2_, CO, CH_4_, N_2_O and NOx emission factors for biomass and peat burning and the references used are also provided in the online supporting material. Total C emissions were computed by using the carbon content in CO_2_, CO and CH_4_; total GHG emissions were computed by using the global warming potentials of CH_4_ (72) and N_2_O (289) over a 20 year time horizon^35^. For comparison with national summaries, total emissions were recalculated using 100 year time horizon global warming potentials. Fuel load, combustion completeness, burned area and gaseous emission results are presented in Table 1.

### Analyses of geospatial data

All the maps presented in this article, and geospatial analyses performed in this study were carried out by the authors of this study using *ArcMap* v10.0 geospatial processing program.

## Author Contributions

D.L.A.G., M.A.S., K.H. and B.L. designed and performed the study and data acquisition. D.L.A.G., M.A.S., E.M., and H.Y. created and analysed the maps. B.L., D.L.A.G., M.A.S., M.W., and M.M. analysed the fire and rainfall datasets. K.H., L.V., and D.L.A.G. quantified the emissions of carbon and GHG. R.D., L.V., R.N., D.M., P.H. and D.S. supervised the work. D.L.A.G., D.S. and S.S. wrote the manuscript, with feedback from all authors.

## Supplementary Material

Supplementary Information Supplementary Figures Results and Methods

## Figures and Tables

**Table 1:** Carbon emissions from fires. Average value ± SE of fuel load (FL), combustion completeness (CC), burned areas (detailed as burned on mineral soils + peat soils for each vegetation cover defined in Fig. 3), emission of carbon dioxide (CO_2_), carbon monoxide (CO), methane (CH_4_), nitrous oxide (N_2_O), mono-nitrogen oxides (NOx), total carbon (C) emission (CO_2_+CO+CH_4_) and total emission of greenhouse gases (GHG) (CO_2_+CH_4_+N_2_O). Total emission of GHG were calculated using 20 (GHG_20YGWP_) and 100 year (GHG_100YGWP_) global warming potentials (GWP) for CH_4_ and N_2_O. The lack of appropriate emission factors for other GHG species prevented their inclusion

	Non-Forest	*Acacia*	Forest	Peat soil	Total
FL (Mg DM ha^−1^)	55.9 ± 10.5	56.0 ±4.4	205.6 ± 48.8	353‡ ± 187	
CC (%)	90 ± 13	90 ±9	38 ± 4		
Burned area (ha)	21,654^m^ + 111,561^p^	119^m ^+ 4,630^p^	2,083^m^ + 9,954^p^	137,044^a^	163,336^b^
CO_2 _(Tg)	10.59 ± 2.55	0.38 ± 0.05	1.49 ± 0.07	82.39 ± 44.35	94.84 ± 44.42
CO (Tg)	0.70 ± 0.19	0.02 ± 0.00	0.10 ± 0.01	10.16 ± 5.47	10.98 ± 5.47
CH_4 _(Tg)	0.05 ± 0.01	0.0016 ± 0.0004	0.01 ± 0.00	1.01 ± 0.54	1.06 ± 0.54
N_2_O (Tg)	0.0013 ± 0.0003	0.00005 ± 0.000008	0.00019 ± 0.00002	-	0.0016 ± 0.0003
NOx (Tg)	0.0107 ± 0.0042	0.0004 ± 0.0001	0.0015 ± 0.0005		0.013 ± 0.004
Total C (Tg)	3.22 ± 0.70	0.12 ± 0.01	0.45 ± 0.02	27.58 ± 12.33	31.37 ± 12.35
GHG_20YGWP_ (Tg CO_2_-eq)	14.26 ± 2.75	0.51 ± 0.06	2.00 ± 0.12	154.83 ± 59.06	171.60 ± 59.13
GHG_100YGWP_ (Tg CO_2_-eq)	11.96 ± 2.56	0.43 ± 0.05	1.68 ± 0.08	103.52 ± 45.78	117.58 ± 45.85

^m^Area burned on mineral soils. ^p^ Area burned on peat soils.

^a^Includes 10,899 ha on peat soils under cloud before fire, and for which previous vegetation cover could not be assessed but for which peat emissions were included; ^b^ Includes 2,436 ha on mineral soil under cloud before fire for which emissions were excluded.

^‡^roduct of fuel load (FL) and combustion completeness (CC).
