# Supplementary material for: Major atmospheric emissions from peat fires in Southeast Asia during non-drought years: evidence from the 2013 Sumatran fires
Source: Sci Rep. 2014 Aug 19;4:6112. doi: 10.1038/srep06112 (PMC4137341; doi:10.1038/srep06112)
Supplement: Supplementary Information — Supplementary Figures Results and Methods [file srep06112-s1.doc]

**Major atmospheric emissions from peat fires in Southeast Asia during non-drought years: evidence from the 2013 Sumatran fires.**

**David L.A. Gaveau^1^, Muhammad A. Salim^1^, Kristell Hergoualc’h^1^, Bruno Locatelli^1,2^, Sean Sloan^3^, Martin Wooster^4^, Miriam E. Marlier^5^, Elis Molidena^1^, Husna Yaem^1^, Ruth DeFries^5^, , Louis Verchot^1,6^, Daniel Murdiyarso^1,7^, Robert Nasi^1^, Peter Holmgren^1^, Douglas Sheil^1,8^**

^1^ Center for International Forestry Research, P.O. Box 0113 BOCBD, Bogor 16000, Indonesia

^2^ UPR BSEF, CIRAD, TA C-105 / D, Campus international de Baillarguet, 34398 Montpellier Cedex 5, France

^3^ Centre for Tropical Environmental and Sustainability Science, School of Marine & Tropical Biology, James Cook University, PO Box 6811, Cairns, QLD 4870, Australia

^4^ King’s College London, Department of Geography, KCL, Strand, London, WC2R 2LS, UK

^5^ Department of Ecology, Evolution, and Environmental Biology, Columbia University, New York, New York 10027, USA

^6^ Center for Environmental Sustainability, Earth Institute, Columbia University. Schermerhorn Extension, 1200 Amsterdam Avenue New York, NY 10027-5557, USA

^7^ Department of Geophysics and Meteorology, Bogor Agricultural University, Indonesia

^8^ Department of Ecology and Natural Resource Management (INA), Norwegian University of Life Science (NMBU), Box 5003, 1432 Ås, Norway

**Supplementary information**

**Supplementary Figures**


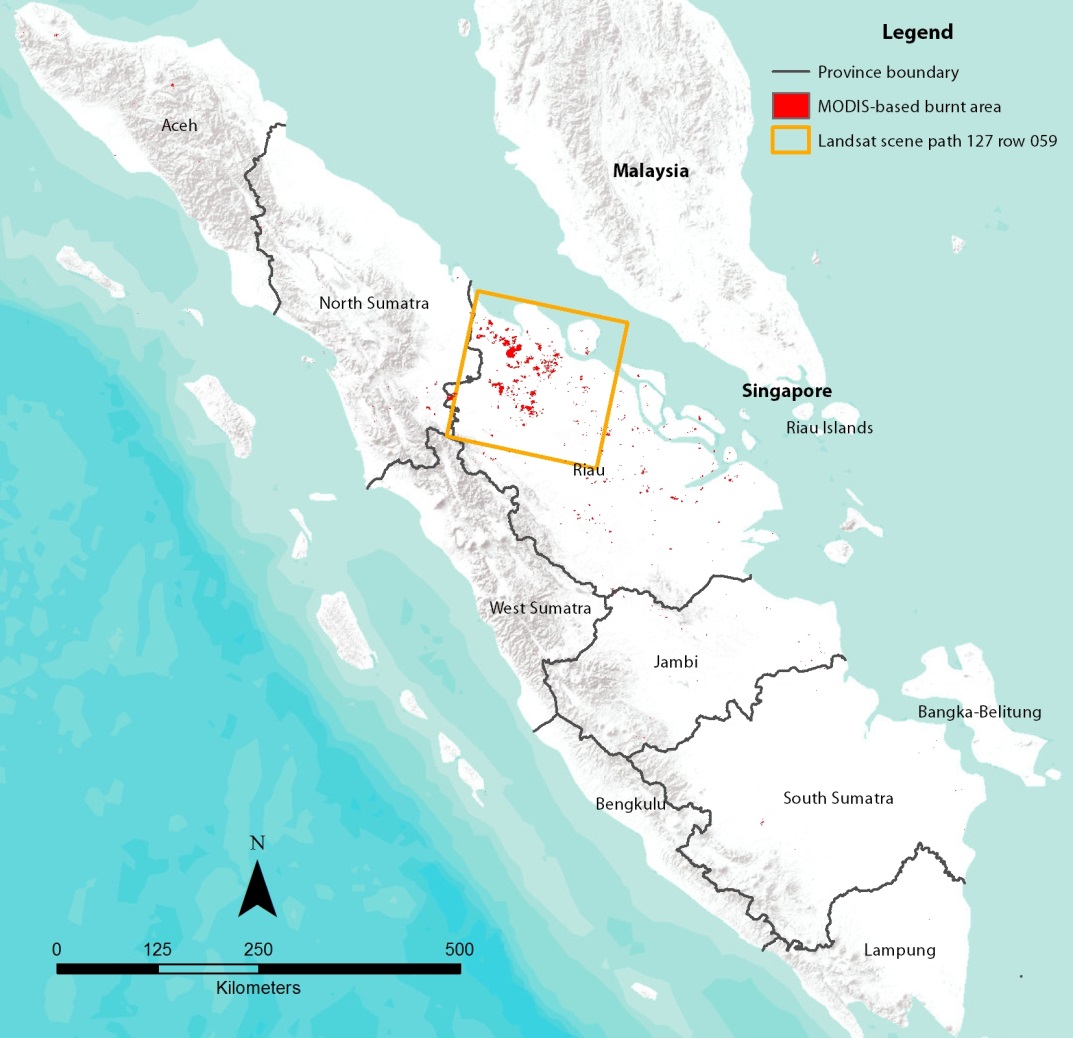


**Supplementary Figure 1** **Burned areas across Sumatra.** An estimated 177,390 ha burned according to the MODIS instruments (TERRA and AQUA satellites; MCD65A1 dataset) in June 2013. Burned areas mapped until July 2013 are included in addition to those in June 2013, since there are uncertainties on the MODIS-derived data of burn, in part due to frequent smoke and cloud cover. Seventy two percent (126,928 ha) of the total area MODIS burned in Sumatra at this time was found in the three-million ha area covered by the single LANDSAT scene (Path/Row: 127/059) we analyse in the province of Riau. We note that MODIS undercounted the area burned by 23% compared with the LANDSAT-based estimate (163,366 ha; Figure 1b), primarily because burns are generally more challenging to detect with the 500 m spatial resolution MODIS imagery compared to the 30 m spatial resolution LANDSAT imagery. Map created using *ArcMap* v10.0 geospatial processing program


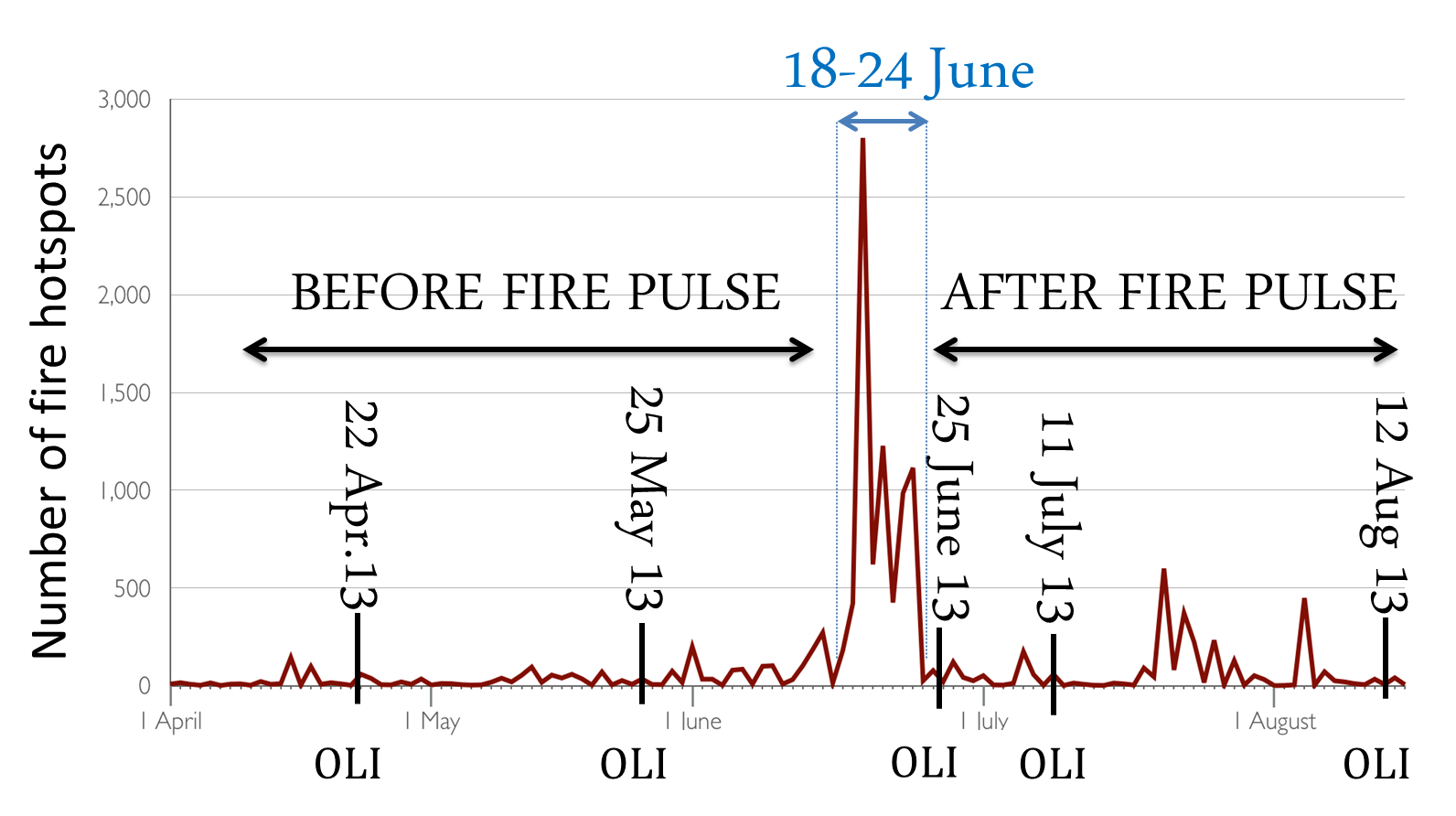


**Supplementary Figure 2** **Daily time series of fire hotspots across Sumatra from 01 April to 15 August 2013.** Hotspots were detected by TERRA and AQUA satellites (the standard MOD14/MYD14 Fire and Thermal Anomalies product available at NASA FIRMS website). This time-series reveals a distinct peak in fire activity during the week of 18-24 June 2013. The dates of LANDSAT OLI imagery used to map burned areas are indicated.


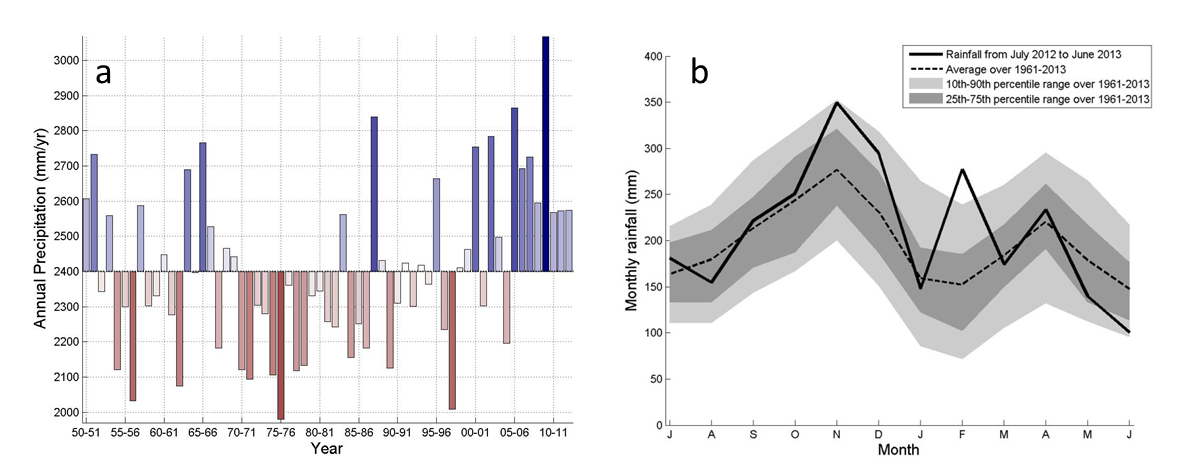


**Supplementary Figure 3 a.** **Annual rainfall in Riau province (Central Sumatra) from 1950 until 2013.** The average is 2,350 mm (period 1961-2013). Our years begin on 01 July and end on 30 June. For example, the last bar to the right represents the annual rainfall from 01 July 2012 until 30 June 2013. It is color-coded blue because it is above the average (2,350 mm). Years that are color-coded red are below the average. **b.** Monthly rainfall in Riau from July 2012 to June 2013 (thick black line) also from PREC/L dataset , 10^th^-90^th^ percentile range over 1961-2013 (light shade area) and 25^th^-75^th^ percentile range (dark shade). Data from NOAA’s PRECipitation REConstruction over Land (PREC/L) dataset .


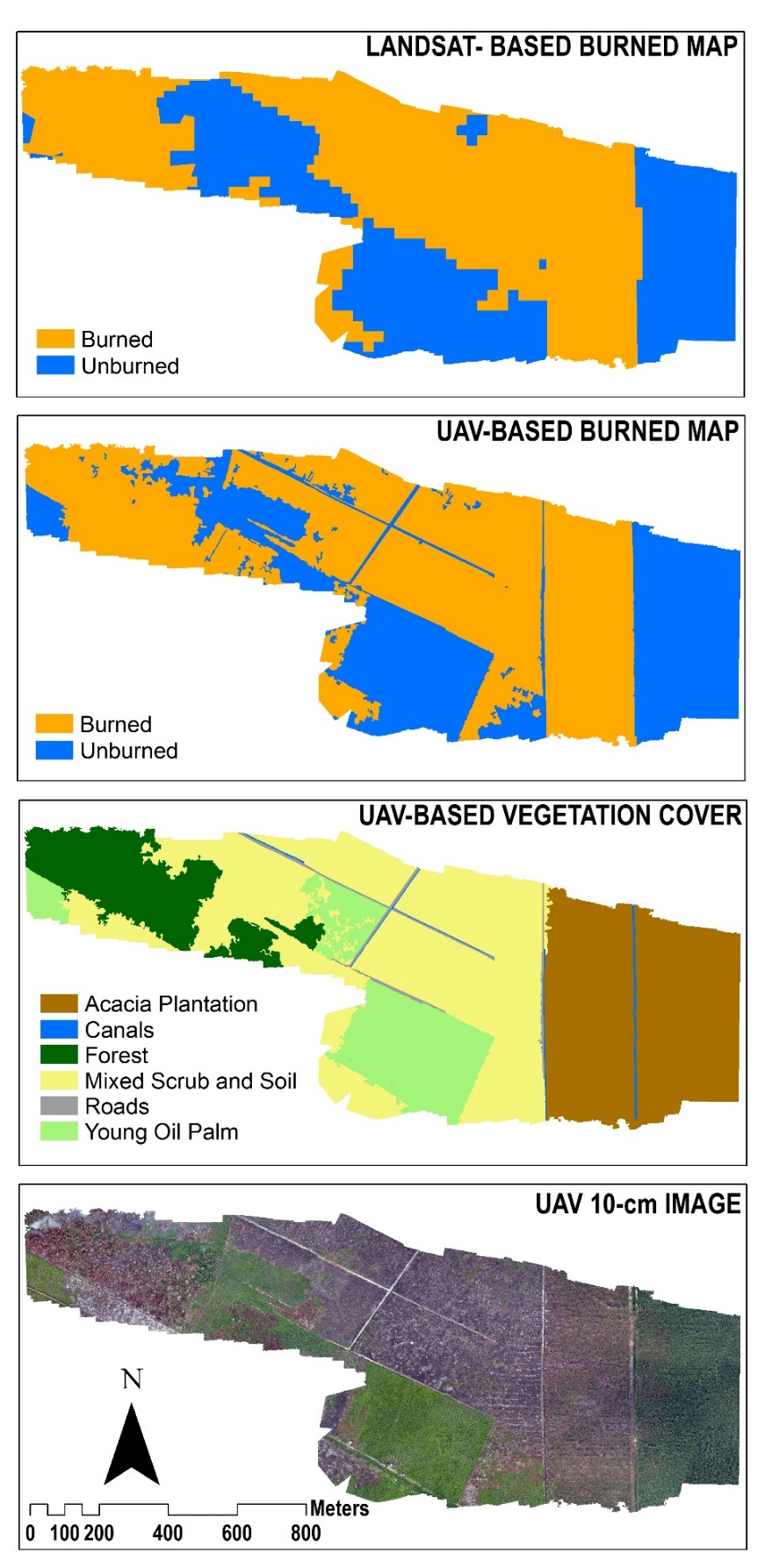


**Supplementary Figure 4Validation of LANDSAT-based maps against UAV.** The UAV-transect for location number 6 (see Fig. 2b), its associated vegetation cover and burned/unburned classification used for validation of corresponding LANDSAT burned/unburned classification. Maps created using *ArcMap* v10.0 geospatial processing program


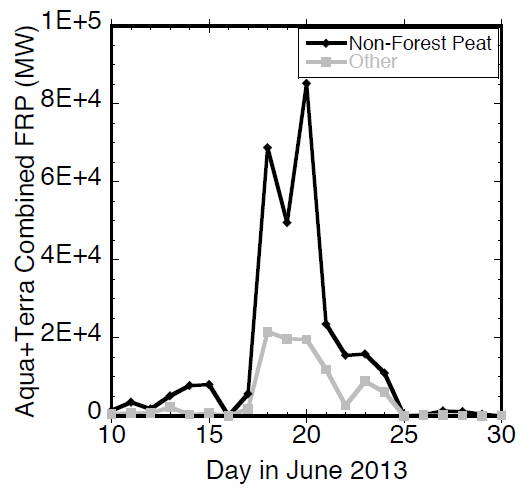


**Supplementary Figure 5** **Fire Radiative Power in the burned areas**. Daily 1-km FRP recorded by the MODIS instruments (TERRA and AQUA satellites; MOD14A1 + MYD14A1 datasets) from 10-30 June 2013 in the LANDSAT-based burned areas mapped in the three million ha area (Fig. 2b).


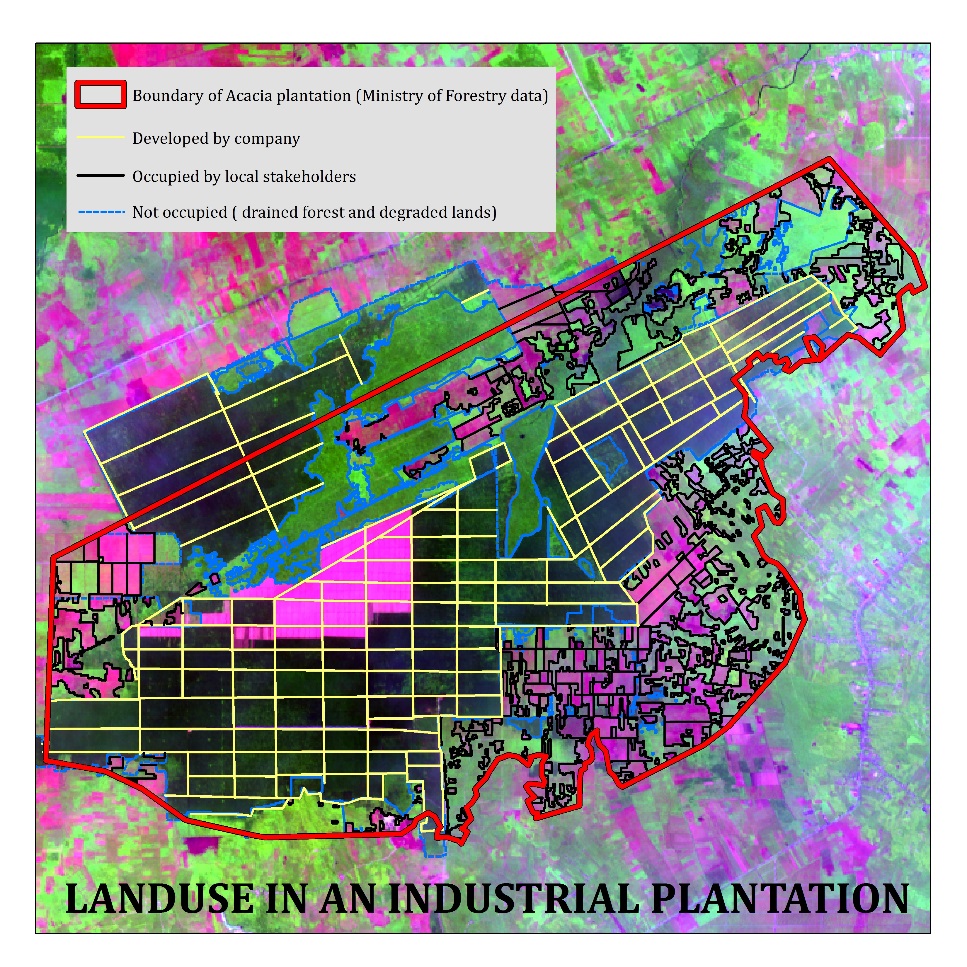


**Supplementary Figure 6 Detailed land ownership in an *Acacia* concession.** Concession boundary obtained from Indonesia’s Ministry of Forestry showing areas developed by companies (the grid-like network in yellow), areas occupied (owned *de facto* but not legally) by small-scale agriculturalists (the clusters of rectangular land parcels of varying shape, size and direction in black), and idle undeveloped lands, like forest and scrub lands (in blue). Map created using *ArcMap* v10.0 geospatial processing program

**
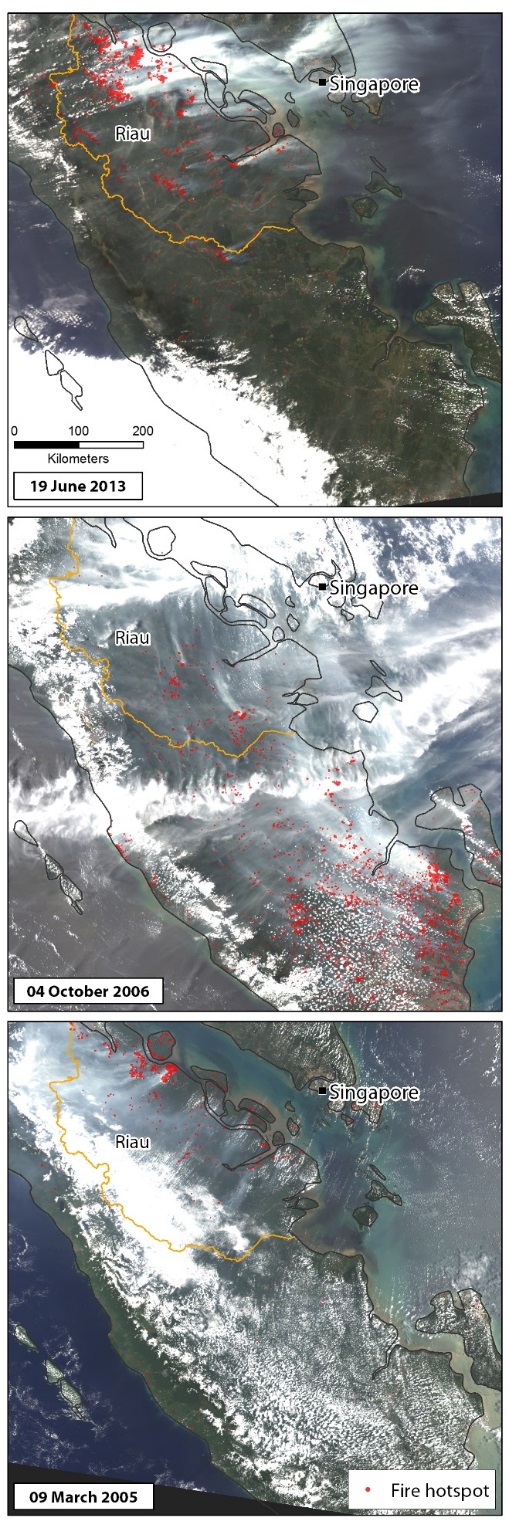
**

**Supplementary Figure 7  Haze formation and direction in Southeast Asia captured by TERRA and AQUA satellites. a,** On 19 June 2013 (a non ENSO year), a smoke plume originated from peatland fires in Riau province, Sumatra. The smoke traveled east because of prevailing south westerly monsoon winds. Singapore’s 24-hr PSI reached a record 246 on 22 June 2013. **b,** On 04 October 2006 (an ENSO year), a smoke plume originated from peat land fires in southeast Sumatra. The smoke travelled north because of prevailing south easterly monsoon winds. The PSI reached 128 on 07 October. **c,** On 05 March 2005 (a non ENSO year), a smoke plume originated from peat land fires in Riau province. The smoke traveled west because of prevailing north easterly winds. The PSI stayed below 60 throughout March. Maps created using *ArcMap* v10.0 geospatial processing program.

**Supplementary results**

**Validation of LANDSAT-based burned areas and pre-fire vegetation cover**

The overall accuracy for the burned/unburned classification is 84.8%. The per-class accuracies of ‘burned’ and ‘unburned’ were similarly high, with user’s accuracy ranging from 82-87%, and producer’s accuracy ranging from 84-85% (Supplementary Table 1, 2)

**Supplementary Table 1**. Confusion matrix of ‘burned’ and ‘unburned’ classification.

| **N=2,088 points** | **UAV Burned** | **UAV Unburned** | **Sum** |
| --- | --- | --- | --- |
| **LANDSAT Burned** | 979 | 144 | 1123 |
| **LANDSAT Unburned** | 173 | 792 | 965 |
| **Sum** | 1152 | 936 | 2088 |

**Supplementary Table 2.** Accuracy results of ‘burned’, and ‘unburned’ classification.

| **Class** | **User Accuracy** | **Producer Accuracy** |
| --- | --- | --- |
| **Burned** | 86.9% | 84.9% |
| **Unburned** | 82.1% | 84.4% |

Overall accuracy (1769/2088)=84.8%

Overall Misclassification Rate: (317 / 2088) = 15.2%

Overall Omission Error: 15.2%

Overall Commission Error: 15.2%

The overall accuracy for the pre-fire ‘Forest’, ‘Non-forest’, ‘*Acacia* Plantation’ classification is 97%. The per-class accuracies of ‘Forest’ and ‘*Acacia*’ and ‘Non-forest’ were similarly high, with user’s accuracy ranging from 94-97%, and producer’s accuracy ranging from 85-99% (Supplementary Table 3, 4)

**Supplementary Table 3.** Confusion matrix of pre-fire ‘Acacia’, ‘Forest’ and ‘Non-Forest’ classification.

| **N=912 points** | **UAV Acacia** | **UAV Forest** | **UAV Non-forest** | **Sum** |
| --- | --- | --- | --- | --- |
| **LANDSAT Acacia** | 66 | 0 | 2 | 68 |
| **LANDSAT Forest** | 0 | 116 | 7 | 123 |
| **LANDSAT Non-forest** | 2 | 20 | 699 | 721 |
| **Sum** | 68 | 136 | 708 | 912 |

**Supplementary Table 4.** Accuracy results of pre-fire ‘Acacia’, ‘Forest’ and ‘Non-forest’ classification.

| **Class** | **User Accuracy** | **Producer Accuracy** |
| --- | --- | --- |
| **Acacia** | 97.0% | 97.0% |
| **Forest** | 94.3% | 85.3% |
| **Non-forest** | 96.9% | 98.7% |

Overall accuracy (881/912) =96.6%

Overall Misclassification Rate: (31 / 912) = 15.2%

Overall Omission Error: 3.4%

Overall Commission Error: 1.7%

**Supplementary methods**

Fire emissions are commonly calculated as the product of burned area, fuel load, combustion completeness and gas-specific emission factor[^3^](#_ENREF_3).

The fuel load is the amount of fuel per unit area that can potentially burn in a fire event. Biome-averaged values, expressed in dry matter unit, were used for the phytomass (above and below-ground biomass and necromass) densities. Phytomass densities in forests and *A. crassicarpa* plantations were taken from a literature review in Southeast Asia[^4^](#_ENREF_4). Phytomass density in the non-forest category was calculated by weighting the density of each existing vegetation classes identified by LANDSAT and UAV imagery (see figure 3): (i) scrub/regrowth and exposed soils; (ii) young oil palm (< 5 year old); (iii) mature closed canopy (> 5 year old) oil palm; (iv) *A. crassicarpa* plantation; and (v) degraded forest by its surface contribution to the category (12 ± 5, 45 ± 7, 31 ± 7, 7 ± 4, 2 ± 1, 3 ± 1%, respectively). Bare land (exposed soils) was considered having no vegetation while that in scrub/regrowth was assessed as one third of the density in the forest. Phytomass densities in above- and belowground biomass and ground cover in oil palm plantations were calculated at each age during the 25 year rotation period using existing models and averaged for the first 5 years and following 6-25 years of the rotation for the opened (35.0.1 ± 6.4 Mg DM ha^-1^) and closed canopy (88.6 ± 3.4 Mg DM ha^-1^) plantations, respectively.

Only a fraction of the available fuel load is consumed during a fire event, and this fraction is represented by the combustion completeness. An average value was adopted for degraded forest[^7^](#_ENREF_7). Combustion completeness is poorly investigated for Indonesian land cover types or tropical plantations in general hence we used best judgment estimates which were assessed from pictures taken by the Umanned Aerial Vehicle after the fire took place. Uncertainty, expressed as standard error, were 10% of the estimate value. Combustion completeness in *Acacia* plantations was assessed at 90 ± 9%. Combustion completeness in the non-forest category was calculated similarly to the phytomass density by weighting the combustion completeness of each existing land use class by its surface contribution to the category. Best judgment combustion completeness were 100 ± 10, 90 ± 9, 100 ± 10, 50 ± 5 in bare land (exposed soils), scrub, opened and closed canopy oil palm plantation, respectively.

The emission factor determines the mass of gas emitted per mass of fuel consumed by fire. Emissions of CO_2_, CO, CH_4_, N_2_O and NOx from phytomass and peat burning were calculated using the emission factors for tropical forest provided in two published works. Standard errors associated with the emissions factors were calculated from the standard deviations provided in[^8^](#_ENREF_8) using a conservative number of studies of two (n = 2). Standard errors of emission factors for which no uncertainty value was given by were set at 10% of the emission factor value. Currently for Sumatran peat we use lab-based emission factors from the single peer-reviewed study that has reported for this fuel[^9^](#_ENREF_9). Very recently a new publication has provided emission factor measures from further tropical peat samples, albeit from Kalimantan rather than Sumatra. For completeness, Table 5 reproduces these Kalimantan peat emissions factors to indicate the potential difference these would make to our GHG calculations. The largest change would be in CH_4_ emissions, which would almost halve, underlining the importance of gaining a more complete picture of the emission factor variations for these types of tropical fuel in future.

**Supplementary Table 5**. Emission factors (kg gas Mg^-1^ DM burned). Values are average ± SE.

|  | CO_2_ | CO | CH_4_ | N_2_O | NOx | |
| --- | --- | --- | --- | --- | --- | --- |
| Vegetation | 1580 ± 63.6 | 104 ± 14.1 | 6.8 ± 1.4 | 0.2 ± 0.02 | | 1.6 ± 0.5 |
| Peat (Sumatra) | 1703 ± 170.3 | 210 ± 21 | 20.8 ± 2.1 | - | - | |
| Peat (Kalimantan) | 1485± 77.4 | 213± 42.1 | 11.7± 3.7 | - | - | |

**References**
